# Accurate and Robust Monocular SLAM with Omnidirectional Cameras

**DOI:** 10.3390/s19204494

**Published:** 2019-10-16

**Authors:** Shuoyuan Liu, Peng Guo, Lihui Feng, Aiying Yang

**Affiliations:** The Key Laboratory of Photonics Information Technology, Ministry of Industry and Information Technology, School of Optics and Photonics, Beijing Institute of Technology, Beijing 100086, China; lsyzge405@163.com (S.L.); yangaiying@bit.edu.cn (A.Y.)

**Keywords:** simultaneous localization and mapping, visual SLAM, map initialization, fisheye cameras, omnidirectional cameras

## Abstract

Simultaneous localization and mapping (SLAM) are fundamental elements for many emerging technologies, such as autonomous driving and augmented reality. For this paper, to get more information, we developed an improved monocular visual SLAM system by using omnidirectional cameras. Our method extends the ORB-SLAM framework with the enhanced unified camera model as a projection function, which can be applied to catadioptric systems and wide-angle fisheye cameras with 195 degrees field-of-view. The proposed system can use the full area of the images even with strong distortion. For omnidirectional cameras, a map initialization method is proposed. We analytically derive the Jacobian matrices of the reprojection errors with respect to the camera pose and 3D position of points. The proposed SLAM has been extensively tested in real-world datasets. The results show positioning error is less than 0.1% in a small indoor environment and is less than 1.5% in a large environment. The results demonstrate that our method is real-time, and increases its accuracy and robustness over the normal systems based on the pinhole model.

## 1. Introduction

In order to complete various tasks, the robot needs to know the location of its environment. The most common method to localize a robot is to process the sensor information to calculate incremental motion. To achieve drift-free localization, the robot needs a map where the localization is known. To solve these problems, visual simultaneous localization and mapping (SLAM), where the main sensor is a camera, has been actively researched in recent years [1,2,3,4,5]. Among different sensor modalities, a monocular camera is low-cost and easy to maintain. The feature distribution of the environments observed by the camera is a key factor in the performance of Visual SLAM. A major limiting factor for the feature distribution is the field-of-view (FoV) of the camera. Especially in sparse-feature or partially non-feature environments, wide FoV cameras can get higher accuracy and better robustness than normal cameras.

However, traditional Visual SLAM can only use the part area of the wide FoV images, because these approaches are designed for the pinhole camera model which projects measurements of 3D points onto a 2D image plane. In practice, even when distortion model is added, the pinhole camera model demonstrates suboptimal performance for a FoV greater than 120°. To reduce the FoV of largely distorted image, the input images are usually cropped in traditional Visual SLAM.

To utilize the full area of the wide FoV images, it is necessary to use a new camera model that is well applied to catadioptric systems and wide-angle fisheye cameras. There are several instances of research [6,7,8,9,10] on camera models for large FoV cameras. Considering the accuracy and computation cost of camera models, we chose the enhanced unified camera model (EUCM) [7] as a projection function.

Since the projection function is changed, a new map initialization method should be proposed. Monocular SLAM needs a process to create an initial map because depth cannot be recovered from a single image. For planar scenes, by using the method of Faugeras et al. [11], the relative camera pose is recovered from a homography matrix. On the other hand, for non-planar scenes, the relative camera pose is recovered from a fundamental matrix which can be computed with the eight-point algorithm [12]. Because these methods are only suitable for the pinhole camera model, we propose a new initialization method based on them for the EUCM.

**Figure 1:**
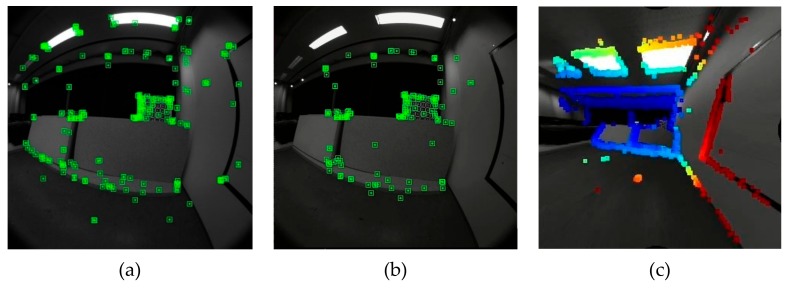
The selected points in a same scene: (**a**) The improved ORB-simultaneous localization and mapping (SLAM) with omnidirectional camera; (**b**) the normal ORB-SLAM; (**c**) Direct Sparse Odometry.

We improve ORB-SLAM [1,2] to make full use of the fisheye images (see Figure 1), so our method can directly use all the information of the input images.

In the experiments, we evaluated the accuracy and run-time of our approach on a benchmark [13] whose image sequences are captured with a wide FoV fisheye lens. We compared our approach to the normal systems based on the pinhole model and demonstrated that our method outperforms the normal methods on benchmark datasets.

The rest of this paper is structured as follows: In Section 2, we first review the related systems for the normal and omnidirectional cameras. In Section 3 and Section 4, we introduce notation and the enhanced unified camera model. In Section 5, we provide an overview of our system, and we analytically derive the Jacobian matrices. In Section 6, we propose a map initialization method and modify a normal relocalization algorithm for fisheye cameras. In Section 7, we quantitatively evaluate the results of our system on public datasets and compare it with the normal systems based on the pinhole model; then we discuss and interpret the results. We open source in https://github.com/lsyads/fisheye-ORB-SLAM.

## 2. Related Works

Over the last decades, many visual SLAM and visual odometry systems have been proposed, such as ORB-SLAM [1,2], LSD-SLAM [4], and direct sparse odometry (DSO) [3]. The three recent examples are the state-of-the-art systems for the normal cameras and points features. ORB-SLAM is an indirect and keyframe-based visual SLAM algorithm based on graph optimization. LSD-SLAM is the first direct visual SLAM method with monocular cameras, which tracks the camera motion, produces a semi-dense map and performs pose graph optimization. DSO is a direct sparse visual odometry algorithm, which combines a fully direct probabilistic model with joint optimization of all model parameters. In addition to points features, Visual SLAM for point-line [14] or point-plane [15] features has been studied for many years. Next, we will discuss the related work on omnidirectional odometry and SLAM.

By using scale-invariant feature transform (SIFT) features and the extended Kalman filter (EKF), several works [16,17] have been proposed to estimate camera localizations for omnidirectional cameras. However, these approaches lack efficient loop closing and relocalization technique, and they are only capable of mapping small work-spaces due to computational limitations.

By using a direct method, D. Caruso et al. [18] proposed a real-time, direct monocular SLAM method based on LSD-SLAM by incorporating the unified camera model [6]. Similarly, H. Matsuki et al. [19] extended DSO for omnidirectional cameras by using the unified camera model as a projection function. This system was the first fisheye-based direct visual odometry which runs in real time. L. Heng et al. [20] presented a semi-direct visual odometry for a fisheye-stereo camera. A direct visual odometry for a fisheye-stereo camera was proposed by P. Liu et al. [21].

Indirect methods are the most popular techniques for SLAM. They proceed in two steps. First, the feature points in the images are extracted and matched. Second, the geometry and camera motion are estimated through the coordinates of corresponding points. J. Li et al. [22] presented a SLAM system based on spherical model for full-view images in indoor environments, but the system was not real-time. S. Wang et al. [23] proposed to extend ORB-SLAM framework by the unified camera model and the semi-dense depth map, whose map initialization method follows the idea from [18], but there was no evaluation on localization accuracy and robustness of the system.

In this paper, we propose an omnidirectional camera extension of ORB-SLAM by using the EUCM as a projection function. This is a real-time robust monocular visual SLAM. Since the projection function is changed, we propose a new map initialization method and modify the normal relocalization algorithm.

## 3. Notation

Throughout the paper, bold lower-case letters (x) represent vectors, and light lower-case letters (*t*) represent scalars. Matrices will be represented by bold upper-case letters (H), and functions (including images) will be represented by light upper-case letters (*I*). Pixel coordinates generally are denoted as $u={\left[u,v\right]}^{T}\in {\mathbb{R}}^{2}$. Point coordinates in 3D are denoted as $x={\left[x,y,z\right]}^{T}\in {\mathbb{R}}^{3}$. ${\left[x\right]}_{\times }$ stands for:

(1) $${\left[x\right]}_{\times }=\left[\begin{array}{ccc} 0 & -z & y\\ z & 0 & -x\\ -y & x & 0 \end{array}\right].$$

The camera orientation and position are represented by R∈SO(3) and $t\in {\mathbb{R}}^{3}$, respectively. They transform a 3D point from the camera coordinate system to the world coordinate system. Traditionally, π stands for a camera projection function and $\pi^{-1}$ is the camera unprojection function.

## 4. Camera Models

### 4.1. Pinhole Model

The pinhole model is the most common camera model. Image points u are computed by projecting measurements of 3D points x onto a 2D image plane. The projection function of the pinhole model is given as

(2) $$u=\left[\begin{array}{c} u\\ v \end{array}\right]=\left[\begin{array}{cc} f_{x} & 0\\ 0 & f_{y} \end{array}\right]\left[\begin{array}{c} x/z\\ y/z \end{array}\right]+\left[\begin{array}{c} c_{x}\\ c_{y} \end{array}\right],$$

where $f_{x},f_{y}$ are the focal lengths, and $c_{x},c_{y}$ are the principal points. The projection function is linear in homogeneous coordinates, so it is the simplest model.

### 4.2. Extended Unified Camera Model

We use the enhanced unified camera model (EUCM) based on the so-called unified camera model [6] for a wide FoV fisheye camera. The projection model does not require additional mapping to model distortions, and it takes just two projection parameters more than a simple pinhole model to represent radial distortions (only one parameter more than the unified model). The unprojection function can be expressed in explicit closed-form.

**Figure 2:**
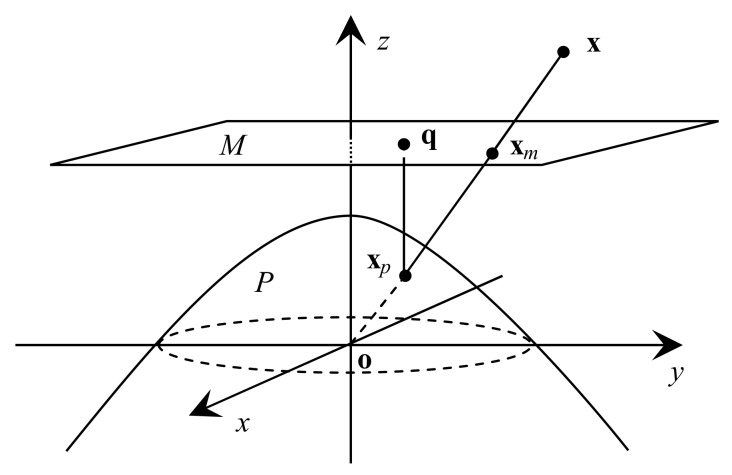
The enhanced unified camera model. z is the optical axis.

As shown in Figure 2, a 3D point x in camera coordinates is first mapped to x*_p_* by projecting it onto a second-order projection surface *P*, then the point x*_p_* is mapped to q by projecting it onto the plane *M* of z=1. The coordinates of the point q are computed as follows:

(3) $$\begin{array}{l} q=\left[\begin{array}{c} x/[\alpha \rho +(1-\alpha )z]\\ y/[\alpha \rho +(1-\alpha )z]\\ 1 \end{array}\right],\\ \rho =\sqrt{\beta (x^{2}+y^{2})+z^{2}}, \end{array}$$

where α∈[0,1] and β>0. The two parameters allow us to better approximate the properties of lenses despite strong distortions. For the fisheye cameras, the EUCM projects on the ellipsoid, where α∈(0.5,1].

**Figure 3:**
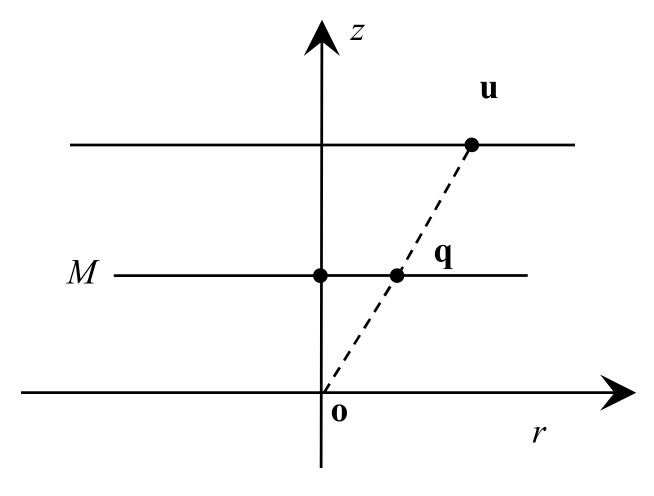
The pinhole camera model.

Finally, the image point u is computed by projecting the point q onto an image plane using the pinhole camera model (see Figure 3). The projection function of a 3D point is given by

(4) $$\pi (x)=u=\left[\begin{array}{c} u\\ v \end{array}\right]=\left[\begin{array}{cc} f_{x} & 0\\ 0 & f_{y} \end{array}\right]\left[\begin{array}{c} x/[\alpha \rho +(1-\alpha )z]\\ y/[\alpha \rho +(1-\alpha )z] \end{array}\right]+\left[\begin{array}{c} c_{x}\\ c_{y} \end{array}\right].$$

The unprojection function is defined as follows:

(5) $$\begin{array}{l} \pi^{-1}(u)={\left[\begin{array}{ccc} m_{x} & m_{y} & m_{z} \end{array}\right]}^{T},\\ m_{x}=\frac{u-c_{x}}{f_{x}},\\ m_{y}=\frac{u-c_{y}}{f_{y}},\\ m_{z}=\frac{1-\beta \alpha^{2}r^{2}}{\alpha \sqrt{1-(2\alpha -1)\beta r^{2}}+(1-\alpha )}, \end{array}$$

where $r^{2}=m_{x}^{2}+m_{y}^{2}$, and if α>0.5, $r^{2}\leq 1/\beta (2\alpha -1)$. $m_{z}$ depends on $m_{x}$ and $m_{y}$. Actually, $\max(m_{z})=1$.

Actually, the unprojection function (5), a ray function, is decided by the coordinates of point x*_p_*, because points x*_p_* and x are on the same line (see Figure 2). Plane *P* where point x*_p_* is located is called *the projection surface*. The function, which solves the coordinates of point x*_p_* from u, is the same as (5):

(6) $$x_{p}={\left[\begin{array}{ccc} m_{x} & m_{y} & m_{z} \end{array}\right]}^{T}.$$

## 5. System Overview

By using the EUCM as a projection function, we develop a real-time robust monocular visual SLAM system for omnidirectional cameras based on ORB-SLAM. The system makes use of ORB features [24], which are based on the FAST keypoint detector and BRIEF descriptor. Not only are they extremely fast in computation and matching, but they also have good invariance in regards of the viewpoint. In order to increase efficiency and accuracy, there are three threads in parallel: tracking, local mapping and loop closing.

### 5.1. Bundle Adjustment

Because Bundle Adjustment (BA) provides a powerful network of matches and good initial guesses, our system uses it to optimize the estimations of camera poses and 3D world points.

There are three kinds of BA in the system: motion-only BA, local BA, and full BA. By minimizing the reprojection error between matched 3D points x in world coordinates and keypoints u, BA optimizes the camera position t, orientation R, and points x. In motion-only BA, it only optimizes camera poses:

(7) $$\begin{array}{l} \{R,t\}=\underset{R,t}{min}\sum_{i\in \chi }\rho (e_{i}^{T}\sum_{i}^{-1}e_{i}),\\ e_{i}=u_{i}-\pi (Rx_{i}+t), \end{array}$$

where χ means the set of all matches, ρ is the robust Huber cost function, and $\Sigma_{i}=\sigma_{i}^{2}I_{2\times 2}$ is the covariance matrix associated to the scale where the keypoint was detected.

In addition to optimizing camera poses, local BA also optimizes points and minimizes the reprojection error in a collection of nearby keyframes. In full BA, all keyframes are optimized to get camera poses and points.

The Levenberg–Marquardt algorithm implementation in g2o [25] is used to solve this minimization problem. In BA, we modified the projection function to EUCM and Jacobian matrices.

### 5.2. Jacobian Matrices

Jacobian matrices are used in the Levenberg–Marquardt algorithm to speed up a bundle adjustment process in the SLAM system, because they are more efficient than numeric or automatic differentiation. A 3D point in world and camera coordinates are represented by $x={\left[x,y,z\right]}^{T}$ and $x^{\prime }={\left[x^{\prime },y^{\prime },z^{\prime }\right]}^{T}$, respectively. The reprojection error (7) is written as *e*. The small changes of camera rotation and position are represented by δR and δt, respectively.

The projection relation is $u=\pi (x^{\prime })$ from (4), and (3) is reduced to $m={\left[\begin{array}{cc} x^{\prime }/(\alpha \rho +(1-\alpha )z^{\prime }), & y^{\prime }/\left(\alpha \rho +(1-\alpha )z^{\prime }\right) \end{array}\right]}^{T}$.

By using the chain rule, the Jacobian matrices of the reprojection errors with respect to the camera rotation, camera position, and 3D points is computed, respectively:

(8) $$J_{R}=\frac{\partial e}{\partial \delta R}=\frac{\partial e}{\partial x^{\prime }}\frac{\partial x^{\prime }}{\partial \delta R}=\frac{\partial e}{\partial x^{\prime }}(-{\left[x^{\prime }\right]}_{\times }),$$

(9) $$J_{t}=\frac{\partial e}{\partial \delta t}=\frac{\partial e}{\partial x^{\prime }}\frac{\partial x^{\prime }}{\partial \delta t}=\frac{\partial e}{\partial x^{\prime }}I=\frac{\partial e}{\partial x^{\prime }},$$

(10) $$J_{x}=\frac{\partial e}{\partial x}=\frac{\partial e}{\partial x^{\prime }}\frac{\partial x^{\prime }}{\partial x}=\frac{\partial e}{\partial x^{\prime }}R,$$

where

(11) $$\begin{array}{l} \frac{\partial e}{\partial x^{\prime }}=-\frac{\partial u}{\partial x^{\prime }}=-\left[\begin{array}{cc} f_{x} & 0\\ 0 & f_{y} \end{array}\right]\frac{\partial q}{\partial x^{\prime }},\\ \frac{\partial q}{\partial x^{\prime }}=\left[\begin{array}{ccc} \begin{array}{c} \frac{1}{\eta }-\frac{\alpha \beta {x^{\prime }}^{2}}{\eta^{2}\rho }\\ -\frac{\alpha \beta x^{\prime }y^{\prime }}{\eta^{2}\rho } \end{array} & \begin{array}{c} -\frac{\alpha \beta x^{\prime }y^{\prime }}{\eta^{2}\rho }\\ \frac{1}{\eta }-\frac{\alpha \beta {y^{\prime }}^{2}}{\eta^{2}\rho } \end{array} & \begin{array}{c} -\frac{x^{\prime }(1-\alpha +(\alpha z^{\prime })/\rho )}{\eta^{2}}\\ -\frac{y^{\prime }(1-\alpha +(\alpha z^{\prime })/\rho )}{\eta^{2}} \end{array} \end{array}\right],\\ \eta =\alpha \rho +(1-\alpha )z^{\prime }. \end{array}$$

The Jacobian matrixes for the EUCM are different from the Jacobian matrixes for the pinhole model, so it is necessary to analytically derive the Jacobian matrices.

### 5.3. Tracking, Local Mapping, and Loop Closing

The tracking is responsible for positioning the camera per frame and deciding when to insert a new keyframe. To get the initial camera pose and initial map points, we first perform a map initialization for omnidirectional cameras, which is explained in detail in Section 6.1. If the initialization is successful, we optimize the pose using *motion-only BA*. Then a local map is acquired. It contains the map points of nearby keyframes. Matches with the local map points are searched by reprojection, then the camera pose and the map points are optimized with all matches. Finally, the tracking thread decides if a new keyframe is inserted. When the tracking is lost, the relocalization module for omnidirectional cameras starts working, which is explained in Section 6.2.

The local mapping processes the new keyframes and executes *the local BA* to optimize camera poses and correct map point positions. New points are created by using a triangulation method which is the same as the triangulation of initialization for omnidirectional cameras.

The loop closing is in charge of searching for loops with every new keyframe. For omnidirectional cameras, this thread is almost the same as the original thread except *the full BA* and projection function, which should be the EUCM instead of the pinhole model.

## 6. Map Initialization and Relocalization Algorithm

### 6.1. Map Initialization Algorithm

The goal of the map initialization is to get the relative pose and triangulate a set of initial map points from matching feature points of two frame. Two geometrical models, the homography matrix assuming a planar scene and the essential matrix assuming a non-planar scene, are computed in parallel. Then a suitable model is automatically selected, and the relative pose is recovered with the selected model.

**Figure 4:**
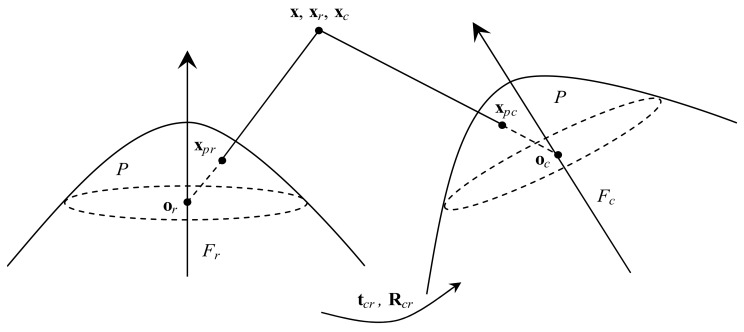
The points $x_{pc}$ and $x_{pr}$ back project to rays, and the intersection of the two rays is the object point x. From the new matches $x_{pc}↔x_{pr}$, we can recover the relative pose $\left[R_{cr}|t_{cr}\right]$ between two frames and triangulate the point x.

The method first extracts ORB features in the current frame $F_{c}$ and reference frame $F_{r}$. It then searches for matches $u_{c}↔u_{r}$. By (6), the coordinates of points $x_{pc}={\left[m_{xc},m_{yc},m_{zc}\right]}^{T}$ and $x_{pr}={\left[m_{xr},m_{yr},m_{zr}\right]}^{T}$ (see Figure 4) are solved from $u_{r}$ and $u_{c}$, respectively, so there are *new matches* $x_{pc}↔x_{pr}$. A point position in world, a current frame and the reference frame homogeneous coordinates are represented by $x={\left[x,y,z,1\right]}^{T}$, $x_{c}={\left[x_{c},y_{c},z_{c},1\right]}^{T}$ and $x_{r}={\left[x_{r},y_{r},z_{r},1\right]}^{T}$, respectively.

As shown in Figure 4, reference frame center o*_r_*, points x*_pr_* and x*_r_* are on the same line, and current frame center o*_c_*, points x*_pc_* and x*_c_* are on the same line. There are two non-zero scale factors $\lambda_{r}$ and $\lambda_{c}$:

(12) $$\begin{array}{l} \lambda_{r}x_{pr}=x_{r}=\left[R_{r}|t_{r}\right]x,\\ \lambda_{c}x_{pc}=x_{c}=\left[R_{c}|t_{c}\right]x, \end{array}$$

where $\left[R_{r}|t_{r}\right]$ and $\left[R_{c}|t_{c}\right]$ are 3×4 matrices which represent camera poses.

#### 6.1.1. The Homography Matrix

The homography matrix is suitable for a planar scene where points x are located. The plane can be set to z=0, so $x={\left[x,y,0,1\right]}^{T}$. By using (12), we get

(13) $$\begin{array}{l} \lambda_{r}x_{pr}=\left[R_{r}|t_{r}\right]{\left[x,y,0,1\right]}^{T}=H_{r}{\left[x,y,1\right]}^{T},\\ \lambda_{c}x_{pc}=\left[R_{c}|t_{c}\right]{\left[x,y,0,1\right]}^{T}=H_{c}{\left[x,y,1\right]}^{T}, \end{array}$$

where $H_{r}$ and $H_{c}$ are 3×3 matrices.

From (13), we get

(14) $$\lambda_{c}x_{pc}=\lambda_{r}H_{c}H_{r}^{-1}x_{pr}=\lambda_{r}H_{cr}x_{pr},$$

where $H_{cr}$ is the homography matrix that can recover the relative pose between two frames.

The scale factors in (14) is eliminated by the cross product:

(15) $$x_{pc}\times (H_{cr}x_{pr})=0.$$

There is no scale factors $\lambda_{r}$ and $\lambda_{c}$ in (15), so all the *new matches points* $x_{pc}↔x_{pr}$ of the two frames satisfy this equation for the same $H_{cr}$. This form (15) will derive a simple linear solution to $H_{cr}$, and it is solved by the normalized direct linear transformation (DLT) as explained in [12] inside a random sample consensus (RANSAC) scheme.

#### 6.1.2. The Essential Matrix

The essential matrix is suitable for a non-planar scene where points x are located. As shown in Figure 4, point $x_{r}$ can be transformed into $x_{c}$ by:

(16) $$x_{c}=\left[R_{cr}|t_{cr}\right]x_{r},$$

where $\left[R_{cr}|t_{cr}\right]$ stands for the relative pose between current frame and reference frame.

From (16), we get the definition equation for the essential matrix $E_{cr}$ in [12]:

(17) $$x_{c}^{T}E_{cr}x_{r}=0,$$

where $E_{cr}={\left[t_{cr}\right]}_{\times }R_{cr}$.

By substituting (12) into (17):

(18) $$\begin{array}{c} \begin{array}{c} {\left(\lambda_{c}x_{pc}\right)}^{T}E_{cr}\left(\lambda_{r}x_{pr}\right)=0. \end{array} \end{array}$$

Because $\lambda_{r}\neq 0$ and $\lambda_{c}\neq 0$, we get

(19) $$\begin{array}{c} \begin{array}{c} x_{pc}^{T}E_{cr}x_{pr}=0. \end{array} \end{array}$$

There are no scale factors $\lambda_{r}$ and $\lambda_{c}$ in (19), so all *new matches points* $x_{pc}↔x_{pr}$ of the two frames satisfy this equation for the same $E_{cr}$. The essential matrix $E_{cr}$ can be solved from (19) by 8-point algorithms [12] inside a RANSAC scheme.

#### 6.1.3. Matrix Selection

The homography matrix $H_{cr}$ and the essential matrix $E_{cr}$ are computed in parallel threads. Then a suitable matrix is automatically selected by a robust heuristic method [2], which chooses a matrix in $H_{cr}$ and $E_{cr}$ with smaller symmetric transfer errors [12].

#### 6.1.4. Motion Recovery and Triangulation Method

We make the motion recovery from the selected matrix, then get the relative pose $\left[R_{cr}|t_{cr}\right]$ between two frames. In the case of the homography matrix, we retrieve 8 motion hypotheses using the method of Faugeras et al. [11]. In the case of the essential matrix, we retrieve 4 motion hypotheses using the singular value decomposition method explained in [12].

We triangulate these hypotheses and select a solution with lower reprojection error. The triangulation method, which is based on the linear triangulation method [12], will be introduced below.

From (12), we can get on *new matches points* $x_{pc}↔x_{pr}$

(20) $$\begin{array}{l} x_{pr}\times (\left[R_{r}|t_{r}\right]x)=0,\\ x_{pc}\times (\left[R_{c}|t_{c}\right]x)=0. \end{array}$$

A cross product of (20) gives three equations for each image point, of which two are linearly independent. Equation (20) can be written as four independent equations:

(21) $$\left[\begin{array}{c} m_{xr}{\left(P_{r}^{3}\right)}^{T}-m_{zr}{\left(P_{r}^{1}\right)}^{T}\\ m_{yr}{\left(P_{r}^{3}\right)}^{T}-m_{zr}{\left(P_{r}^{2}\right)}^{T}\\ m_{xc}{\left(P_{c}^{3}\right)}^{T}-m_{zc}{\left(P_{c}^{1}\right)}^{T}\\ m_{yc}{\left(P_{c}^{3}\right)}^{T}-m_{zc}{\left(P_{c}^{2}\right)}^{T} \end{array}\right]x=0,$$

where $P=\left[R|t\right]$ and $P^{i}$ are the rows of P. Equation (21) is solved by using the singular value decomposition, then the world coordinates of x are obtained.

### 6.2. Relocalization Algorithm

The relocalization model starts working when the tracking is lost. For omnidirectional cameras, the normal relocalization model based on ORB-SLAM is modified. The relocalization algorithm performs first an ORB matching with each candidate keyframes. The method then performs RANSAC iterations of the EPnP algorithm [26] to find a camera pose supported by enough inliers.

Because the EPnP algorithm is only suitable for the pinhole model, and the image surface must be plane, we modified the relocalization model for the EUCM. In order to meet the conditions of the EPnP algorithm, we map first an image point u to a point x*_p_*, then we map the point x*_p_* to a point x*_m_* which is the intersection of the ray ox*_p_* and the z=1 plane (see Figure 2). The coordinates of x*_m_* are computed by using (5):

(22) $$x_{m}={\left[\begin{array}{ccc} m_{x}/m_{z} & m_{y}/m_{z} & 1 \end{array}\right]}^{T}(m_{z}\neq 0).$$

The point x*_m_* meets the conditions of the EPnP algorithm. The value of $m_{z}$ is not allowed to be too small to improve the accuracy of the EPnP algorithm. In our system, we let $\min(m_{z})=0.1$, and there are very few points of $m_{z}<0.1$. Basically, we use the coordinates of x*_m_* instead of image points u in the EPnP algorithm.

## 7. Results and Discussion

**Figure 5:**
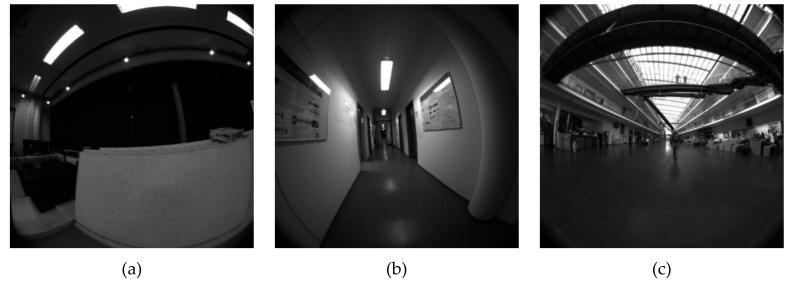
Sample images in the datasets: (**a**) room; (**b**) corridor; (**c**) magistrale.

We performed an experimental evaluation of our omnidirectional ORB-SLAM in terms of accuracy and robustness on the TUM VI benchmark [13]. The datasets provide camera images at 20Hz with a 195 degrees FoV fisheye lens. The image resolution is 512×512. We obtained the camera intrinsic parameters by using the Kalibr calibration toolbox [27] with the original checkerboard sequence. We used 3 types of the datasets: room, corridor, and magistrale. Figure 5 shows the images from the datasets. The room datasets are the sequences in the room with Motion Capture system, where ground-truth poses are available for the entire trajectory. The corridor datasets are sequences in the corridor and several offices. The magistrale datasets are sequences in the large hall. The ground-truth poses of the above two datasets are available for the start and end segments in the same room with Motion Capture system.

We provided a quantitative comparison between our system and other systems, including the normal ORB-SLAM and DSO. Since the normal ORB-SLAM and DSO use the pinhole model as the projection function, we used the Kannala-Brandt model [8] with 8 parameters to rectify the key points in ORB-SLAM and rectify and crop the image in DSO. We evaluated both algorithms in an Intel Core i5-8300H notebook computer with 16GB RAM.

### 7.1. Accuracy and Robustness Comparison

We measured the absolute translational root mean square error (RMSE) between the estimated and the ground-truth position. The estimated position is computed on all keyframes and is aligned with the ground-truth trajectory data. We get the scale factor by least square method where we minimize the difference between the vector length of the estimated position and the truth position. To facilitate a fair comparison: In the room datasets, the alignment is performed for all keyframes; in the corridor and magistrale datasets, the alignment is only performed on the start segments; and we disable loop detection in the corridor and magistrale datasets except the room datasets which are used to demonstrate the loop detection for fisheye cameras. We have run each sequence 5 times in these three systems and listed median results of each time, to account for the randomness of the multithreading system. The results shown in Table 1 demonstrate our system outperforms the other systems in accuracy and robustness. The accuracy of our system is typically below 5 cm in small indoor rooms, below 15 cm in corridors and of a few meters in the large hall, because the length is longer and longer, and the image overlap between frames is smaller and smaller. The drift computed by percentage is below 0.1% in indoor rooms and corridors, and it is also below 1.5% in the large hall. In Table 1, there are many “LOST” in the normal systems, when the camera rotates and moves too fast, and the illumination variation is big.

**Figure 6:**
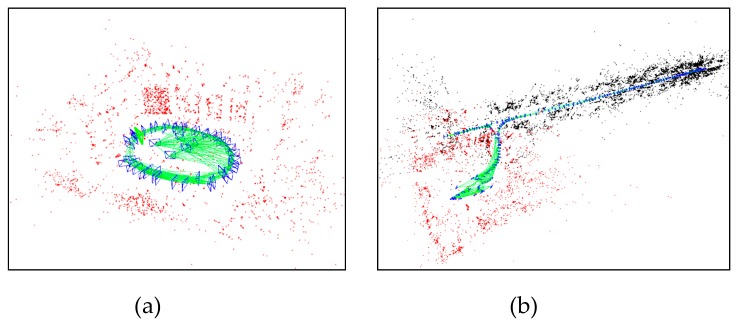
Examples of reconstructed map of the omnidirectional ORB-SLAM. Red points are active points, black points are old points, and blue rectangles are keyframes. Dataset: (**a**) room2; (**b**) corridor4.

**Figure 7:**
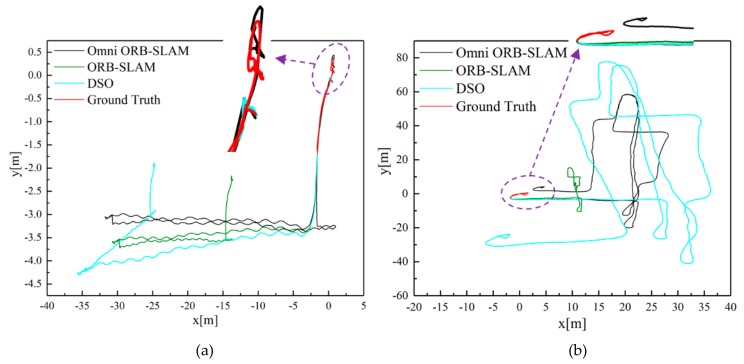
Estimated trajectories. The ground truth is available for the start and end segments in the same room. Dataset: (**a**) corridor4; (**b**) magistrale2.

Some representative visual results and estimated trajectories are shown in Figure 6 and Figure 7. Figure 6a shows a room map whose points are almost active due to wide FoV of fisheye camera; Figure 6b is a map of the same room and a corridor, where datasets start and end in the same room. As shown in Figure 7, the error of all systems is very small in the start segment, but our system suffers less drift than other systems in the end segment because constraints between keyframes are stronger in our system. Due to the wider FOV and the well-maintained scale, our system performs better than the normal ORB-SLAM and DSO.

**Figure 8:**
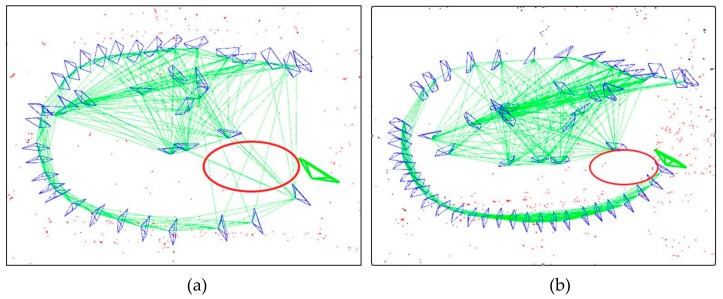
In the red circle, the green lines represent the constraints between keyframes (blue). Our system has more lines than the normal ORB-SLAM. (**a**) The omnidirectional ORB-SLAM; (**b**) The normal ORB-SLAM. (Dataset: room2).

A major advantage of fisheye cameras is that more features can be observed in an input image. Figure 1 shows our system can observe more points in a frame than the normal ORB-SLAM. The image overlap between frames is also bigger, so the constraints between keyframes is stronger in our system, as shown in the red circle of Figure 8.

### 7.2. Timing Measurement

Table 2 shows the measured average time over the datasets for the tracking thread. These results demonstrate our system is real-time and each frame can be tracked at least 30Hz. The time cost of our system is slightly larger than the normal ORB-SLAM, because the normal ORB-SLAM needs time to rectify and crop the key points, and our system needs not rectifying and cropping the input key points but needs more time to compute on the projection function. Obviously, DSO needs more time to rectify and crop the input images and more time to compute the map points because the direct method.

### 7.3. Relocalization Experiments

We performed two relocalization experiments on the datasets. In the experiments, we built a map with the first 40 s of the two sequences *room2* and *room3*, and performed global relocalization with every successive frame. We performed the same experiment with the normal ORB-SLAM for comparison. Table 3 shows the number of the initial map keyframes and the recall. Our system recalls better and needs less keyframes to create the initial map than the normal ORB-SLAM because more features can be observed in an input image by using fisheye cameras.

## 8. Conclusions

We proposed a real-time monocular SLAM system for fisheye cameras. We first incorporated the enhanced unified camera model within the ORB-SLAM. Our system can use the full area of the images, even with strong distortion, except relocalization algorithm, which can also use more points than the normal algorithms because the points on the projection surface of EUCM are directly used. We analytically derived the Jacobian matrices to speed up the bundle adjustment process. A map initialization method was proposed for fisheye cameras. We proved *the new matches points* satisfy the homography matrix assuming a planar scene and the essential matrix assuming a non-planar scene to motion recovery and triangulation method. And we modified the relocalization algorithm for omnidirectional cameras. Then, we compared the performance of our omnidirectional ORB-SLAM and the normal systems on the public datasets, and demonstrated our method yields better performance in accuracy and robustness than the normal methods. In future work, we will extend our method with point features specially for fisheye cameras, inverse depth, and IMU.

## Figures and Tables

**Table 1:** Comparison of translation root mean square error (RMSE). DSO = direct sparse odometry.

Sequences	Keyframe Trajectory RMSE (m) (“X” means LOST)			Length (m)
	DSO	Normal ORB-SLAM	Omnidirectional ORB-SLAM	
corridor1	X	X	0.1252	305
corridor2	X	X	0.1349	322
corridor3	X	X	0.1360	300
corridor4	17.6904	11.2678	0.1196	114
corridor5	X	X	0.1159	270
magistrale1	X	20.3902	11.5939	918
magistrale2	13.4714	10.0507	4.7239	561
magistrale3	X	X	7.4427	566
magistrale4	X	X	9.9546	688
magistrale5	X	X	5.2586	458
room2	0.2868	0.0623	0.0466	142
room3	0.1806	0.0497	0.0446	135

**Table 2:** Mean Timing Results (ms).

Sequences	DSO	Normal ORB-SLAM	Omnidirectional ORB-SLAM
corridor4	137.96	24.6	30.3
magistrale2	125.17	29.1	30.8
room2	267.56	28.6	29.3

**Table 3:** Results for the Relocalization Experiments. KF = Kalman filter.

System	KFs of Initial Map	Recall of Relocalization (%)
*room2, 2081 frames to relocalize*		
Normal ORB-SLAM	75	84.1
Omni ORB-SLAM	52	88.8
*room3, 2020 frames to relocalize*		
Normal ORB-SLAM	93	61.6
Omni ORB-SLAM	61	76.1
